# Hypoxia-immune-related microenvironment prognostic signature for osteosarcoma

**DOI:** 10.3389/fcell.2022.974851

**Published:** 2022-12-12

**Authors:** Wenshuo Zhang, Pang Lyu, Darja Andreev, Yewei Jia, Fulin Zhang, Aline Bozec

**Affiliations:** Department of Internal Medicine 3-Rheumatology and Immunology, Friedrich-Alexander-University Erlangen-Nürnberg (FAU) and Universitätsklinikum Erlangen, Erlangen, Germany

**Keywords:** osteosarcoma, hypoxia, immune status, prognosis, bioinformatic analysis

## Abstract

**Introduction:** Increasing evidences have shown that hypoxia and the immune microenvironment play vital roles in the development of osteosarcoma. However, reliable gene signatures based on the combination of hypoxia and the immune status for prognostic prediction of osteosarcoma have so far not been identified.

**Methods:** The individual hypoxia and immune status of osteosarcoma patients were identified with transcriptomic profiles of a training cohort from the TARGET database using ssGSEA and ESTIMATE algorithms, respectively. Lasso regression and stepwise Cox regression were performed to develop a hypoxia-immune-based gene signature. An independent cohort from the GEO database was used for external validation. Finally, a nomogram was constructed based on the gene signature and clinical features to improve the risk stratification and to quantify the risk assessment for individual patients.

**Results:** Hypoxia and the immune status were significantly associated with the prognosis of osteosarcoma patients. Seven hypoxia- and immune-related genes (BNIP3, SLC38A5, SLC5A3, CKMT2, S100A3, CXCL11 and PGM1) were identified to be involved in our prognostic signature. In the training cohort, the prognostic signature discriminated high-risk patients with osteosarcoma. The hypoxia-immune-based gene signature proved to be a stable and predictive method as determined in different datasets and subgroups of patients. Furthermore, a nomogram based on the prognostic signature was generated to optimize the risk stratification and to quantify the risk assessment. Similar results were validated in an independent GEO cohort, confirming the stability and reliability of the prognostic signature.

**Conclusion:** The hypoxia-immune-based prognostic signature might contribute to the optimization of risk stratification for survival and personalized management of osteosarcoma patients.

## Introduction

Osteosarcoma is highly aggressive malignant bone tumor predominately occurring in children and young adults (Rothzerg et al., 2020). Due to rapid progression and high metastasis rate, it often results in poor prognosis (Niu et al., 2020). The 5-year survival rate of patients with local or metastatic osteosarcoma is about 70% or 20%, respectively (Negri et al., 2019). Most treatment strategies for osteosarcoma involve surgical resection, chemotherapy, and radiotherapy (Jawad et al., 2011). Although significant progress has been made in available therapies, the overall survival of osteosarcoma patients has unfortunately not obviously improved since the last 3 decades (Anderson, 2016; Pierrevelcin et al., 2020). Therefore, identification of new effective prognostic signatures and therapeutic targets might improve osteosarcoma prognosis and treatment.

Hypoxia is a major factor involved in the occurrence and development of tumors, which is related to the imbalance between rapid proliferation of tumor cells and insufficient oxygen supply (Harris, 2002; Ruan et al., 2009). Evidence indicates that hypoxia is associated with aggressive tumor phenotypes and treatment resistance (Bertout et al., 2008; Vooijs et al., 2008; Chang and Erler, 2014). Hypoxia-inducible factor 1α (HIF-1α) is the main molecular transcriptional mediator in response to hypoxia (Semenza, 2014). Studies revealed that HIF-1α upregulation significantly correlated with metastasis and poor prognosis of osteosarcoma patients (Ouyang et al., 2016; Zhang et al., 2018; Pierrevelcin et al., 2020). Notably, hypoxia can modulate the development and function of infiltrating immune cells, thereby affecting the state of the tumor immune microenvironment (Palazón et al., 2012). Studies have shown that hypoxia can regulate the function of myeloid-derived suppressor cells (MDSCs) and redirect their differentiation toward tumor-associated macrophages (TAMs) in the tumor microenvironment (Corzo et al., 2010). Hypoxia can also reduce the activation level of cytotoxic T lymphocytes (CTLs) in a HIF-1α-dependent manner, resulting in immunosuppression and evasion of immune detection (Barsoum et al., 2014). Importantly, infiltrating immune cells, which account for the primary non-tumor components in the tumor immune microenvironment, are closely related to the prognosis and treatment of osteosarcoma (Buddingh et al., 2011; Gomez-Brouchet et al., 2017).

The hypoxia-immune-related gene signature has been already associated with the overall survival in multiple types of tumors, as renal cell carcinoma (Gui et al., 2021), hepatocellular carcinoma (Hu et al., 2020), gastric cancer (Liu et al., 2020), and triple-negative breast cancer (Zheng et al., 2020). These studies have demonstrated that the hypoxia-immune-based signatures possess high prognostic potential and clinical guidance values. However, there is a lack of investigation regarding the interaction of hypoxia and immune cells and its prognostic potential in osteosarcoma, which will be the main research question of this manuscript.

Here, we have comprehensively analyzed the hypoxia and immune status of osteosarcoma to explore the effect of hypoxia and immune interaction on overall survival of osteosarcoma patients. Moreover, we have established a novel prognostic signature based on hypoxia-immune-related genes and performed molecular experimental verification. Our present research may provide new strategies for targeted therapy of osteosarcoma and promote individual-based treatment of patients.

## Materials and methods

### Data collection

All data about osteosarcoma in this study were obtained from the Therapeutically Applicable Research To Generate Effective Treatments (TARGET) (https://ocg.cancer.gov/programs/target/) and Gene Expression Omnibus (GEO) (http://www.ncbi.nlm.nih.gov/geo/) (Barrett et al., 2005) databases. The datasets involved in this study meet the following criteria: 1) samples diagnosed as osteosarcoma; 2) samples with complete clinical information including survival time, vital status, age and sex; 3) sample size in the dataset with more than fifty individuals.

In our research, ninety five samples from the TARGET database were defined as the training cohort, and fifty three samples from the GSE21257 dataset were used as the validation cohort. The flowchart of this study is shown in Supplementary Figure S1.

### Identification of hypoxia status and hypoxia-related DEGs

First, Gene Set Enrichment Analysis (GSEA) was performed on hypoxia pathway between metastatic and non-metastatic osteosarcoma samples in the TARGET dataset. To determine whether hypoxia is an essential characteristic of osteosarcoma compared to healthy control, GSE99671 was used as an additional independent enrichment dataset (Ho et al., 2017). Then, Based on the single-sample gene set enrichment analysis (ssGSEA) method and 200 hypoxia hallmark genes from the Molecular Signatures database (MSigDB) (Liberzon et al., 2011), we calculated the hypoxia enrichment score to predict the hypoxia status. Maximally selected rank statistics were applied by using R packages “survival” and “survminer” to identify the best optimal cutoff value to divide patients. According to the best cutoff value, patients with high hypoxia scores were assigned to hypoxia-high group, while the other samples were ranged to hypoxia-low group. Furthermore, expression changes analysis with reference to HIF-1 signaling pathway-related genes were conducted to explore the difference between hypoxia-high and hypoxia-low groups. These genes were retrieved from the Kyoto Encyclopedia of Genes and Genomes (KEGG) database, including 15 genes related to “increased oxygen delivery” and 13 genes involved in “reduced oxygen consumption”. The R package “limma” was used to identify differentially expressed genes (DEGs) between the two groups. Genes with *p* < 0.05 and an absolute value of log2 (fold change) > 0.5 were considered as hypoxia-related DEGs.

### Identification of immune infiltration status and immune-related DEGs

The newly developed Estimation of Stromal and Immune cells in MAlignant Tumours using Expression data (ESTIMATE) algorithm was applied to calculate scores reflecting the level of infiltrating immune and stromal cells in the tumor microenvironment (Yoshihara et al., 2013). The osteosarcoma patients were attributed to immune-high group and immune-low group based on the median of the immune score. Next, we used ssGSEA method based on the 29 immune signatures, including diverse immune cell types, immune-related functions, and immune-related pathways to validate the effect of immune grouping and to picture a clustering heat map. Besides, the CIBERSORT deconvolution algorithm was applied to accurately determine the composition of 22 immune cells of all osteosarcoma samples (Chen et al., 2018), and the difference of immune grouping was validated again. The DEGs between the immune-high group and immune-low group were identified by R package “limma”. Genes with *p* < 0.05 and an absolute value of log2 (fold change) > 0.5 were considered as immune-related DEGs.

### Division of the groups based on hypoxia and immune status

The identification of the hypoxia and immune status of each patient has been described above. All samples were further labeled with two-dimension contributions and divided into four groups, including hypoxia-high/immune-low group, hypoxia-low/immune-high group, hypoxia-high/immune-high group and hypoxia-low/immune-low group. R packages “survival” and “survminer” were used to carry out a survival analysis for these four groups. The hypoxia-immune-related DEGs were obtained through the overlap between hypoxia-related DEGs and immune-related DEGs by Venn analysis. To understand the biological functions and pathway enrichment of the hypoxia-immune-related DEGs, Gene Ontology (GO) functional analysis and KEGG pathway enrichment analysis were conducted by applying R package “clusterProfiler”.

### Construction and verification of the hypoxia-immune-based prognostic signature in osteosarcoma

The Least Absolute Shrinkage and Selection Operator (Lasso) analysis and the stepwise Cox proportional hazards regression model were conducted using R package “survival” and “glmnet” to construct a hypoxia-immune-based gene signature. The risk score was established by including normalized gene expression values weighted by their Cox coefficients as follows:

$$risk\;score=\sum_{i=1}^{n}\left(coefficient\;i*expression\;of\;signature\;gene\;i\right)$$

Based on the risk score, we computed the best optimal cutoff value to stratify patients into high- and low-risk groups. The Kaplan-Meier (K-M) method was used to draw survival curves, and the log-rank test was carried out to evaluate differences in overall survival between high- and low-risk groups. Univariate and multivariate Cox regression analyses were performed to explore the independent prognostic value of the gene signature. Time-dependent receiver-operating characteristic (ROC) curves were used to verify the performance of the risk signature by comparing the prediction efficiency with the clinical features. With the help of area under the curve (AUC) values and Cox regression analysis, we compared our prognostic signature with other published osteosarcoma models generated by Fu Y et al. (Fu et al., 2021) and Wu F et al. (Wu et al., 2021). A webserver, GEOexplorer (Hunt et al., 2022), was applied to integrate the TARGET and GSE21257 datasets, and the integrated dataset was used as an additional validation dataset.

### Establishment and calibration of a nomogram

A nomogram was built to quantitatively evaluate the survival probabilities of osteosarcoma based on the result of the multivariate Cox regression analysis. Some clinical parameters, such as age, gender, primary tumor site, and metastasis, as well as the risk score were employed to construct the nomogram utilizing the R packages “rms” and “survival”. Calibration curves were drawn to estimate the divergence between the predicted and actual survival probabilities. Time-dependent ROC analysis was used to determine whether our established nomogram was suitable for clinical use.

### Cell culture, hypoxia treatment and transfection

Human osteoblast cell line hFOB1.19 was kindly provided by Prof. Jutta Ries, Department of Oral and Maxillofacial Surgery, Friedrich-Alexander University Erlangen-Nürnberg (FAU) and cultured in DMEM/F12, GlutaMAX Supplement supplemented with 10% fetal bovine serum (Thermo Scientific). Human osteosarcoma cells Saos-2 and 143B were kindly provided by Prof. Thomas Brabletz, Department of Experimental Medicine 1, Nikolaus-Fiebiger-Center for Molecular Medicine, Friedrich-Alexander-University Erlangen-Nürnberg and maintained in Dulbecco’s Modified Eagle’s Medium (DMEM) with 10% fetal bovine serum. Saos-2 and 143B cells were cultured under normoxic (21% O_2_) or hypoxic (1% O_2_) conditions for 8 h. Specific shRNAs against BNIP3 (shBNIP3) and negative control (shNC) were obtained from Origene. Saos-2 and 143B cells were transfected with shBNIP3 or shNC using Lipofectamine 3,000 Transfection Reagent (Thermo Scientific) according to the manufacturer’s instructions. Saos-2 and 143B cells were collected for RNA and protein extraction following 48 h of incubation with specific shRNAs.

### RNA isolation and quantitative real-time polymerase chain reaction (RT-PCR)

RNA extraction was performed with Trizol reagent (Invitrogen) and complementary DNA was synthesized by using the High-Capacity cDNA Reverse Transcription Kit (Thermo Scientific) according to the manufacturer’s instructions. RT-PCR was performed using the SYBR Select Master Mix (Thermo Scientific) on the QuantStudio 6 Flex Real-Time PCR System (Thermo Scientific) with the primers listed in Table 1. ACTB was used as an internal control and the 2^−ΔΔCt^ method was used for data analysis.

**TABLE 1:** List of primers.

Primer pair name	Sequence (5′- 3′)
BNIP3 (For)	TCA​GCA​TGA​GGA​ACA​CGA​GCG​T
BNIP3 (Rev)	GAG​GTT​GTC​AGA​CGC​CTT​CCA​A
SLC38A5 (For)	GCC​ATA​GCT​CTG​ATC​CTG​CTT​G
SLC38A5 (Rev)	ATG​CGG​AGG​TAG​AAG​ATG​CTG​G
SLC5A3 (For)	GCC​AGT​ACC​ATA​TTC​ACC​CTC​G
SLC5A3 (Rev)	CAT​CTC​CAC​GAT​GAT​TGG​CAC​C
CKMT2 (For)	CTG​GTG​ACG​AGG​AGT​CCT​ATG​A
CKMT2 (Rev)	TCC​GTT​GTG​TGC​TTC​ATC​ACC​C
S100A3 (For)	CAA​ATA​CAA​GCT​CTG​CCA​GGC​G
S100A3 (Rev)	TCG​CAG​TCC​TTG​TTG​GTG​TCC​A
CXCL11 (For)	AAG​GAC​AAC​GAT​GCC​TAA​ATC​CC
CXCL11 (Rev)	CAG​ATG​CCC​TTT​TCC​AGG​ACT​TC
PGM1 (For)	TGA​TGG​ACG​CGA​GCA​AAC​TGT​C
PGM1 (Rev)	ATG​TCC​TCC​ACA​CTC​TGC​TTG​C
ACTB (For)	CAC​CAT​TGG​CAA​TGA​GCG​GTT​C
ACTB (Rev)	AGG​TCT​TTG​CGG​ATG​TCC​ACG​T

### Western blotting

Cultured hFOB1.19, Saos-2, and 143B cells were washed twice with PBS and homogenized into extraction buffer (8 M urea, 10% glycerol, 1% SDS, 10 mM Tris-HCl pH 6.8, protease inhibitor complete (Roche), 1 mM Sodium-Vanadate). Protein concentration was determined with a BCA Protein Assay Kit (Thermo Scientific). An equal amount of protein samples were separated by SDS-PAGE and transferred onto PVDF transfer membranes (Thermo Scientific). Then, membranes were blocked with 5% w/v skim milk, incubated with primary antibodies against BNIP3 (1:1,000; 68091-1-Ig; Proteintech), SLC38A5 (1:1,000; 28102-1-AP; Proteintech), SLC5A3 (1:1,000; 21628-1-AP; Proteintech), CKMT2 (1:1,000; 13207-1-AP; Proteintech), S100A3 (1:1,000; 12343-1-AP; Proteintech), PGM1 (1:1,000; 15161-1-AP; Proteintech), CXCL11 (1:1,000; MAB672-SP; R&D Systems) and β-actin antibody (1:5,000; NB-600; Sigma) overnight at 4 °C. Afterward, Anti-Mouse IgG (H + L), HRP Conjugate (1:5000; W4021; Promega) or Anti-Rabbit IgG (H + L), HRP Conjugate (1:5000; W4011; Promega) was applied for hatching the membranes for 2 h. The membranes were exposed to enhanced chemiluminescence (ECL) solution and the western blot bands were quantified using ImageJ software.

#### Cell proliferation and invasion assay

Transfected osteosarcoma cells were cultured in 96-well plates with a cell density of 5000 cells per well. A total of 10 μL Cell Counting Kit-8 solution (CCK8; HY-K0301; MedChemExpress) was added at 24 and 48 h, respectively. The optical density (OD) of each well was measured using a microplate reader at 450 nm.

After transfection, 500 cells were seeded in a 12-well dish and cultured for 2 weeks. Colonies were fixed and stained using 0.5% crystal violet. Colonies of more than 50 cells were counted under a dissecting microscope.

Transfected cells were seeded in six-well plates and grew until they reached full confluence. Scratches were made using a 10 μL pipette tip. The changes of scratches at 6 h were observed under a microscope. ImageJ software was used to calculate the area change of the scratches.

### Statistical analysis

All analyses were performed with R version 4.1.0 (https://www.r-project.org) and its appropriate packages. Data were analyzed with standard statistical tests as appropriated. *p* values less than 0.05 were considered statistically significant. *p < 0.05, **p < 0.01, ***p < 0.001, ****p < 0.0001.

## Results

### Hypoxia status and hypoxia-related DEGs in osteosarcoma

**FIGURE 1:**
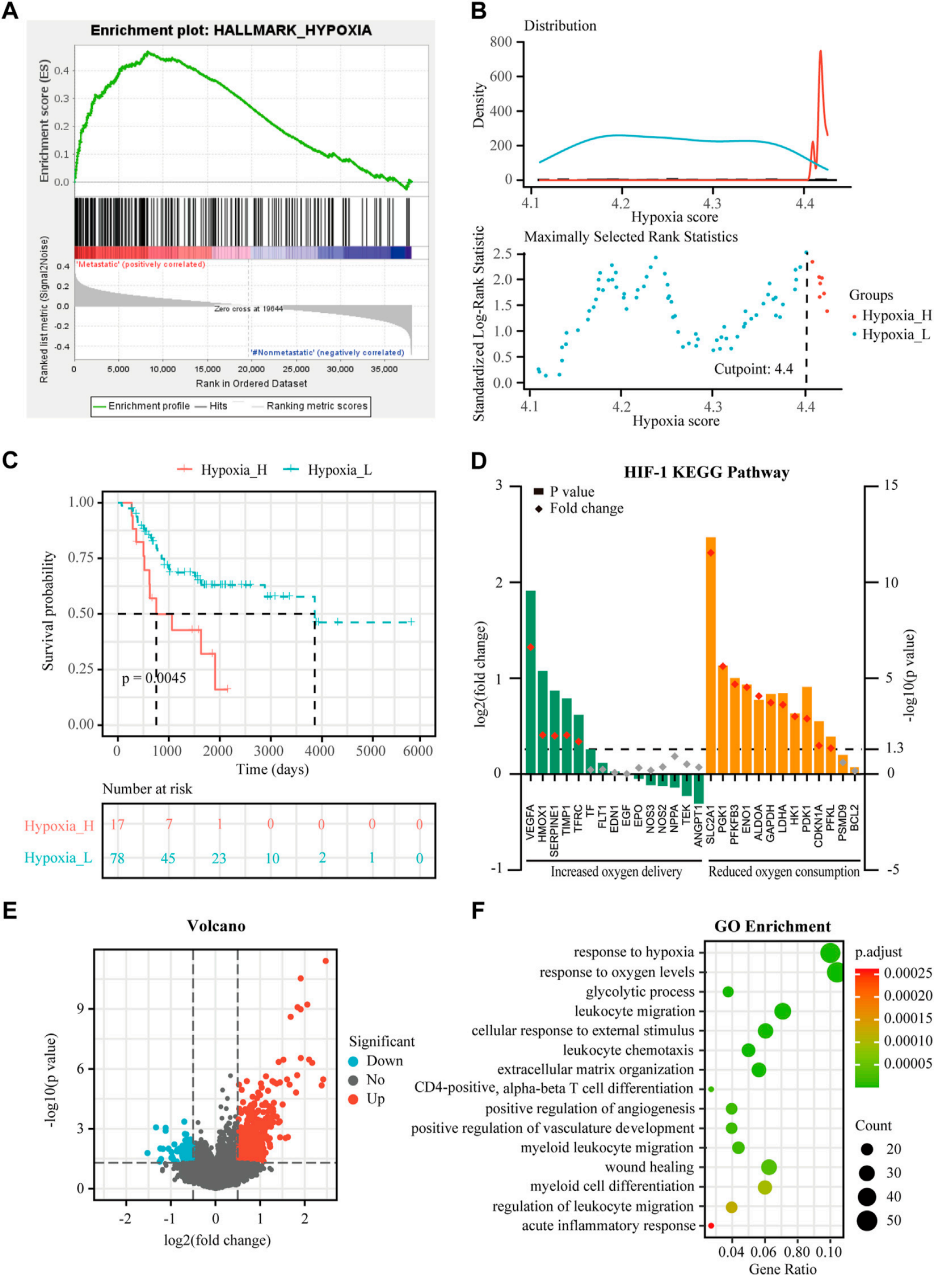
Identification of hypoxia status and hypoxia-related differentially expressed genes (DEGs) in osteosarcoma. **(A)** Gene Set Enrichment Analysis (GSEA) of hypoxia between metastatic and non-metastatic osteosarcoma in the TARGET dataset. **(B)** Histogram of the density distribution of the hypoxia score for hypoxia-high and hypoxia-low groups divided by the optimal cutoff. Scatter plot of the standardized log-rank statistic value for each corresponding hypoxia score cutoff. **(C)** Kaplan-Meier (K-M) plot of overall survival for patients in hypoxia-high and hypoxia-low groups in the TARGET dataset. **(D)** Expression changes (hypoxia-high *versus* hypoxia-low) of target genes involved in HIF-1 KEGG pathway in the TARGET dataset. **(E)** Volcano plot showing the DEGs between hypoxia-high and hypoxia-low groups in the TARGET dataset. **(F)** Gene Ontology (GO) analysis of DEGs between hypoxia-high and hypoxia-low groups in the TARGET dataset.

The training cohort contained 23 metastatic and 72 non-metastatic osteosarcoma samples from the TARGET database. The clinical information of all patients is shown in Table 2. GSEA analysis indicated that metastatic samples exhibited a significant hypoxia enrichment signature compared to non-metastatic samples (NES 1.67and NOM *p*-value 0.046) (Figure 1A). The hypoxia pathway was enriched in osteosarcoma samples when compared to healthy controls (NES 1.50 and NOM *p*-value 0.030) (Supplementary Figure S2). With the list of hypoxia hallmark genes (n = 200), the hypoxia enrichment score of each osteosarcoma patient was quantified by ssGSEA to delineate the hypoxia status. The best cutoff value of “4.4” was determined based on maximally selected rank statistics (Figure 1B). By means of the cutoff value, the hypoxia-high group and hypoxia-low group were divided, containing respectively 17 and 78 patients. K-M survival analysis demonstrated that the prognosis for patients with a high level of hypoxia is significantly worse than for those with a low hypoxia level (log rank test, *p* < 0.05) (Figure 1C). Moreover, we explored the gene expression changes of the HIF-1 signaling pathway, which contained 15 genes involved in “increased oxygen delivery” and 13 genes related to “reduced oxygen consumption”. As shown in Figure 1D, 16 genes (SLC2A1, VEGFA, PGK1, PFKFB3, ENO1, ALDOA, GAPDH, LDHA, HK1, PDK1, HMOX1, TIMP1, SERPINE1, TFRC, CDKN1A, and PFKL) were significantly overexpressed in the hypoxia-high group (*p* < 0.05). According to the above findings, we could define two specific groups among osteosarcoma patients that are associated with the hypoxia status.

**TABLE 2:** The clinical information of osteosarcoma patients in the training cohort from TARGET database.

Characteristics	Whole cohort (n = 95)	Low risk (n = 73)	High risk (n = 22)
Age (year)			
≤16	56 (0.588)	42 (0.575)	14 (0.636)
>16	39 (0.411)	31 (0.425)	8 (0.364)
**Gender**			
Female	40 (0.421)	28 (0.384)	12 (0.545)
Male	55 (0.579)	45 (0.616)	10 (0.455)
**Metastasis**			
Yes	23 (0.242)	1 (0.014)	22 (1.000)
No	72 (0.758)	72 (0.986)	0
**Vital status**			
Alive	57 (0.600)	50 (0.685)	7 (0.318)
Dead	38 (0.400)	23 (0.315)	15 (0.682)
**Primary tumor sites**			
Leg/foot	83 (0.874)	68 (0.932)	15 (0.682)
Arm/hand	7 (0.073)	5 (0.068)	2 (0.091)
Pelvis	4 (0.042)	0	4 (0.182)
Other	1 (0.011)	0	1 (0.045)
**Hypoxia status**			
High	17 (0.179)	10 (0.137)	7 (0.318)
Low	78 (0.821)	63 (0.863)	15 (0.682)
**Immune status**			
High	47 (0.495)	43 (0.589)	4 (0.182)
Low	48 (0.505)	30 (0.418)	18 (0.818)
**Risk group**			
High	22 (0.232)	0	22 (1.000)
Low	73 (0.768)	73 (1.000)	0

Expression profiles were compared between the hypoxia-high and hypoxia-low groups, and 688 DEGs related to hypoxia were obtained (Figure 1E). Enrichment analysis showed upregulated genes in the hypoxia-high group were enriched in the pathways “response to hypoxia”, “response to oxygen levels”, and in several biological processes that are associated with immunological functions, such as “leukocyte migration” and “myeloid cell differentiation” (Figure 1F). Interestingly, the DEGs between hypoxia-high and hypoxia-low groups were also enriched in different immune-related responses, implying an interconnection between the immune state and hypoxia state.

### Immune infiltration status and immune-related DEGs in osteosarcoma

**FIGURE 2:**
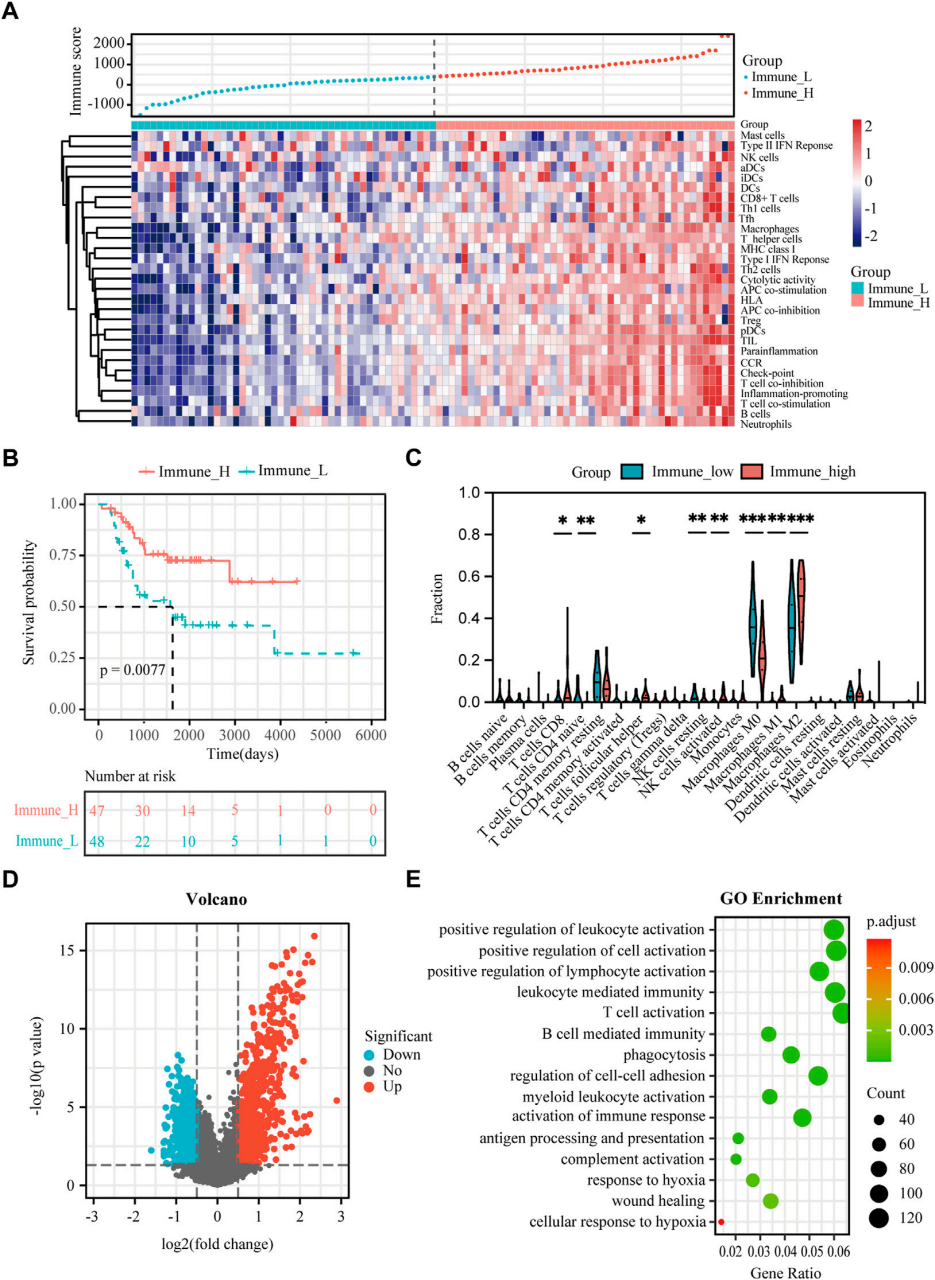
Analysis of immune status and immune-associated DEGs in osteosarcoma. **(A)** Enrichment levels of 29 immune signatures in the immune-high and immune-low groups in the TARGET dataset. **(B)** K-M plot of overall survival for patients in immune-high and immune-low groups in the TARGET dataset. **(C)** Comparison of 22 infiltrating immune cells between immune-low and immune-high groups in the TARGET dataset. **(D)** Volcano plot showing the DEGs between immune-high and immune-low groups in the TARGET dataset. **(E)** GO analysis of DEGs between immune-high and immune-low groups in the TARGET dataset. Asterisks mark statistically significant difference. *p < 0.05, **p < 0.01, ***p < 0.001.

The immune score of each patient in the TARGET dataset was calculated by ESTIMATE to identity the overall level of infiltrating immune cells. According to the median value of the immune score, 95 osteosarcoma patients were divided into immune-high (n = 47) and immune-low groups (n = 48) (Figure 2A). To verify the practicability of immune grouping, 29 immune signatures representing different types of immune cells and corresponding pathways were analyzed. Figure 2A represents the heat map of different activities and abundances of immune cells, pathways and functions in the immune-high group and immune-low group. K-M curves showed that the high immune score was significantly associated with improved survival (log rank test, *p* < 0.05) (Figure 2B). Next, CIBERSORT algorithm was further employed to evaluate infiltration of different immune cells. As showed in Figure 2C, immune-high group showed higher infiltration degrees of CD8^+^ T cell, T follicular helper cells, activated NK cells, M1-like macrophages and M2-like macrophages, while the immune-low group showed higher infiltration degrees of naive CD4^+^ T cells, resting NK cells and M0 (non-activated) macrophages.

The immune-related DEGs were obtained by comparing the gene expression between immune-high and immune-low groups. Altogether, 2,847 DEGs were identified (Figure 2D). The GO enrichment analysis showed that “positive regulation of leukocyte activation”, “positive regulation of cell activation”, and “positive regulation of lymphocyte activation” represent the main biological processes that are associated with the immune status (Figure 2E). It is worth noting that the immune-related DEGs were also enriched in “response to hypoxia”.

Taken together, the immune grouping could properly reflect the different immune status of osteosarcoma patients, highlighting the infiltration patterns of immune cells, which are associated with the survival rate.

### Hypoxia-immune-related DEGs in osteosarcoma

**FIGURE 3:**
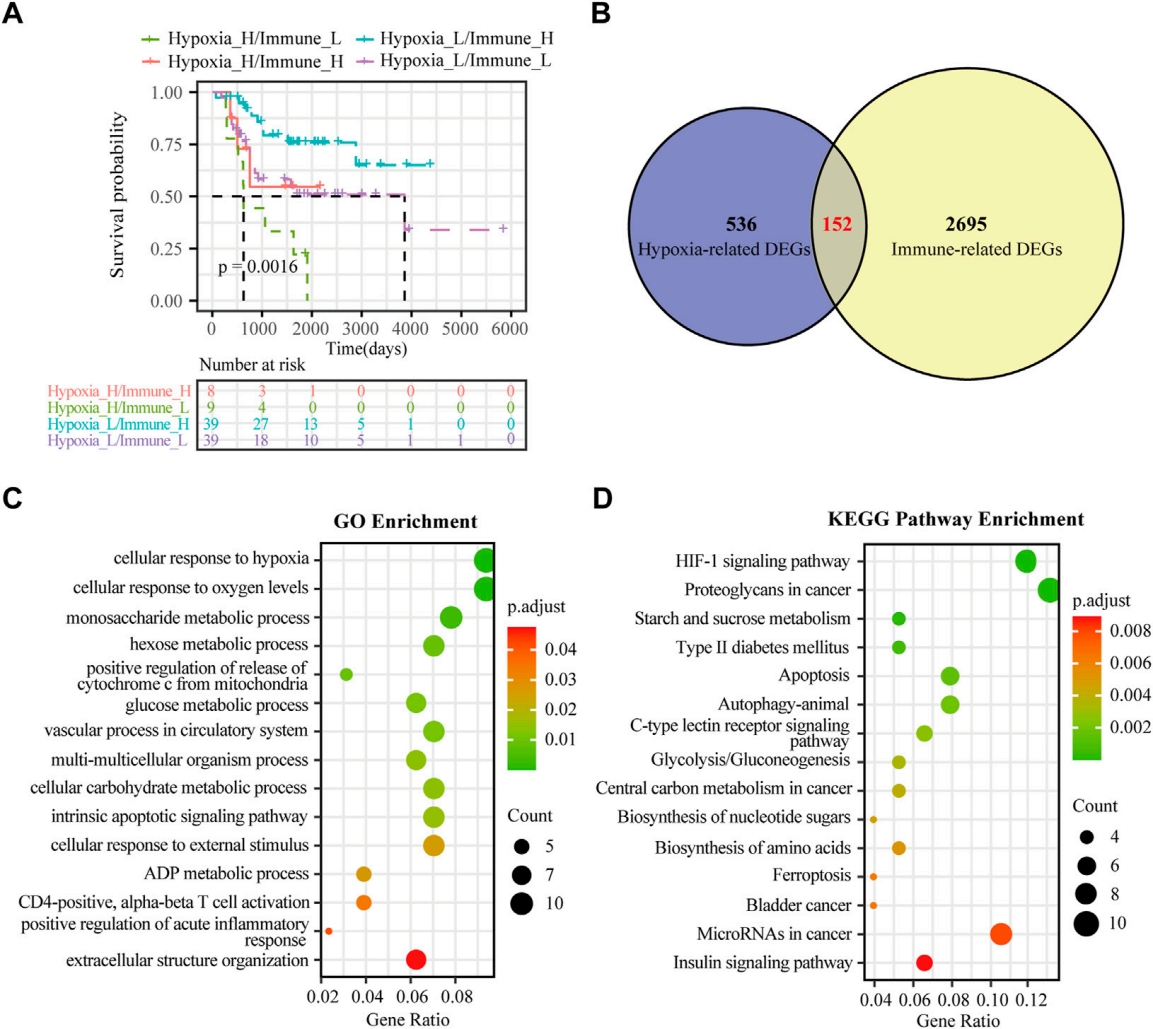
Identification of hypoxia-immune-related DEGs in osteosarcoma. **(A)** K-M plot of overall survival for patients in hypoxia-high/immune-low, hypoxia-low/immune-high, hypoxia-high/immune-high and hypoxia-low/immune-low group in the TARGET dataset. **(B)** Venn diagrams showing overlaps of hypoxia-related DEGs and immune-related DEGs in the TARGET dataset. **(C)** GO analysis for hypoxia-immune-related DEGs in the TARGET dataset. **(D)** KEGG analysis for hypoxia-immune-related DEGs in the TARGET dataset.

Although hypoxia as well as the immune status within the osteosarcoma microenvironment was individually correlated to patients’ overall survival, the effects of hypoxia and immune interaction on the prognosis of osteosarcoma patients remain to be identified. We further considered hypoxia and the immune status together by combining them into a two-dimensional index. To do so, patients were divided into four groups: hypoxia-high/immune-low, hypoxia-low/immune-high, hypoxia-high/immune-high and hypoxia-low/immune-low group. The survival analysis showed significant differences among these four groups, wherein patients in the hypoxia-low/immune-high group had the best prognosis, while those in the hypoxia-high/immune-low group yield the worst survival (log rank test, *p* < 0.01) (Figure 3A). As hypothesized, a more severe hypoxia status and a lower level of immune cell infiltration may lead to a worse prognosis. This provides a hint on an inverse association between the effects of hypoxia and immune cell infiltration in the context of osteosarcoma prognosis.

To determine the DEGs related to hypoxia and immune status, the hypoxia-related DEGs were intersected with the immune-related DEGs. Altogether, 152 overlapping genes were obtained for subsequent analyses (Figure 3B). We further conducted GO enrichment analysis and KEGG pathway analysis to ascertain potential functions of these hypoxia-immune-related DEGs. As depicted in Figure 3C, these DEGs were mainly enriched in pathways that are related to metabolic alteration in response hypoxia, such as “cellular response to hypoxia”, “cellular response to oxygen levels”, “monosaccharide metabolic process”, and “hexose metabolic process”. The KEGG pathway analysis showed that the hypoxia-immune-related DEGs may also be involved in certain cancer or metabolism-related pathways, including “HIF-1 signaling pathway”, “proteoglycans in cancer”, and “starch and sucrose metabolism” (Figure 3D).

### Establishment and evaluation of the hypoxia-immune-based prognositic signature in osteosarcoma

**FIGURE 4:**
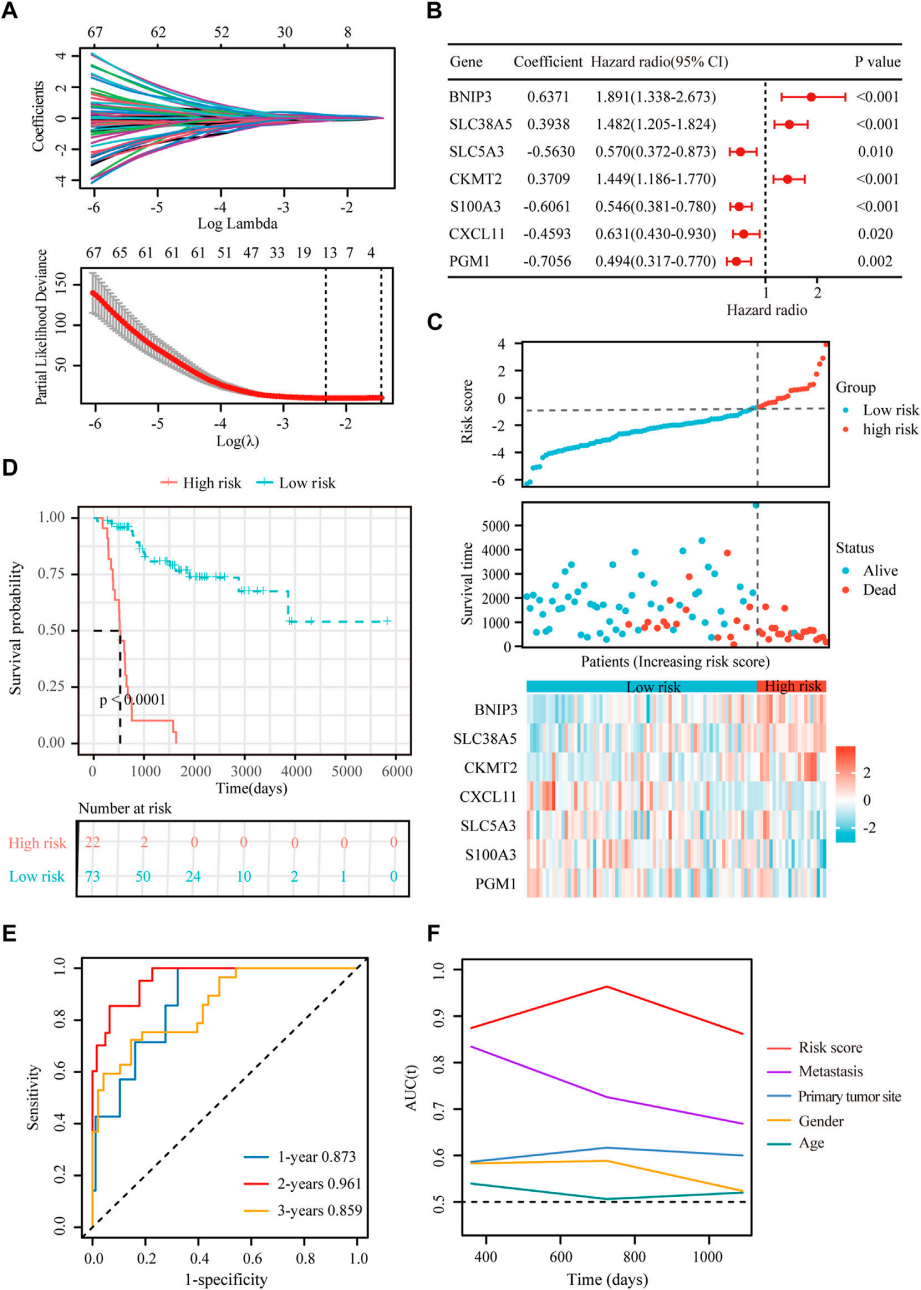
Construction of a hypoxia-immune-related prognostic signature. **(A)** Lasso coefficient profiles of the most relevant prognostic genes (upper panel) and ten-fold cross validation for tuning parameter selection in the Lasso model (lower panel) in the TARGET dataset. **(B)** Forest plots of seven DEGs with *p* < 0.5 by stepwise Cox proportional hazards regression model from the TARGET dataset. **(C)** Risk score, survival status and expression of the seven signature genes in the training cohort from TARGET database. **(D)** K-M curve of overall survival for patients in the high-risk and low-risk groups in the training cohort from TARGET database. **(E)** Receiver operating characteristic (ROC) curve for 1-, 2-, and 3-year overall survival in patients from TARGET dataset. **(F)** Comparisons of AUC values among different clinical characteristics of patients in the TARGET dataset.

To obtain the hypoxia-immune-related prognostic DEGs, Lasso regression analysis was employed to narrow the range of genes. As shown in Figure 4A, the number of independent variable coefficients gradually increased to zero with the gradual increase of lambda. Ten-fold cross validation was used to build the model, and the confidence interval was under each lambda. Thus, we selected 12 DEGs at lambda = 0.0980 as candidate genes. The 12 DEGs were BCL2 Interacting Protein 3 (BNIP3), Solute Carrier Family 38 Member 5 (SLC38A5), Stanniocalcin 2 (STC2), Phosphoglucomutase 1 (PGM1), CFAP20 Domain Containing (CFAP20DC), Galectin 1 (LGALS1), Creatine Kinase, Mitochondrial 2 (CKMT2), C-X-C Motif Chemokine Ligand 11 (CXCL11), Solute Carrier Family 5 Member 3 (SLC5A3), Formin Like 1 (FMNL1), S100 Calcium Binding Protein A3 (S100A3) and T Cell Receptor Alpha Constant (TRAC). To optimize the gene signature and identify only the most predictive DEGs, a stepwise Cox proportional hazards regression model was used, which resulted in a final set of 7 DEGs (BNIP3, SLC38A5, SLC5A3, CKMT2, S100A3, CXCL11 and PGM1) (Figure 4B). Using the expression levels of 7 DEGs and the corresponding coefficients derived from the stepwise Cox proportional hazards regression model, we estimated the risk score for each patient: risk score = (0.6371 * BNIP3 expression) + (0.3938 * SLC38A5 expression) + (-0.5630 * SLC5A3 expression) + (0.3709 * CKMT2 expression) + (-0.6061 *S100A3 expression) + (-0.4593 * CXCL11 expression) + (-0.7056 * PGM1 expression). The distribution of the risk score in the TARGET dataset is shown in Figure 4C. As based on the best optimal cutoff value of the risk score, patients were assigned to high-risk (n = 22) and low-risk (n = 73) groups (Table 2). Results from the K-M survival analysis indicated that the overall survival of patients in the high-risk group was significantly lower than the one of patients in the low-risk group (log-rank test, *p* < 0.0001) (Figure 4D).

To evaluate the extensive applicability of our gene signature, we analyzed the prognostic value of the gene signature in osteosarcoma patients with different clinical features (age, sex and metastatic status). As summarized in Supplementary Figures S3A–F, this gene signature effectively discriminated high-risk patients with poor prognosis in different subgroups (log-rank test, *p* < 0.01), demonstrating a high prognostic value of this genes signature in osteosarcoma.

Besides, univariate and multivariate Cox regressions were conducted to analyze the relationships among gene signature, the clinical features and the overall survival of patients with osteosarcoma. Results from the Cox analysis demonstrated that the risk score was significantly correlated with the overall survival of patients in the training cohort (Table 3). This suggested that the calculated risk score might serve as an independent risk factor for the overall survival in osteosarcoma.

**TABLE 3:** Comparisons between the risk score and other four variables, including age, metastasis, gender, and primary tumor site in the TARGET dataset using univariate and multivariate Cox regression analyses.

**Variable**	Univariate cox regression				Multivariate cox regression			
	HR	Lower 95% CI	Upper 95% CI	*p*-Value	HR	Lower 95% CI	Upper 95% CI	*p*-Value
Risk score	2.72	2.08	3.55	<0.001	2.56	1.93	3.39	<0.001
Age	0.98	0.92	1.05	0.55	1.02	0.96	1.08	0.49
Metastasis	3.81	1.99	7.29	<0.001	2.91	1.40	6.02	<0.01
Gender	1.03	0.54	1.98	0.93	1.06	0.52	2.13	0.88
Primary tumor site	2.57	1.63	4.04	<0.001	1.42	0.84	2.39	0.19

In addition, we conducted ROC curve analysis to evaluate the sensitivity and specificity of the prognostic signature. The AUC values for the 1-, 2-, and 3-year survival rates were 0.873, 0.961 and 0.859, respectively (Figure 4E), suggesting a good predictive value for the prognosis of osteosarcoma patients. Compared with other clinical features, including metastasis and primary tumor site, the ROC curve analysis indicated that the risk score was more accurate than the other tested clinical features for the 1, 2- and 3-year survival prediction (Figure 4F). In comparison with other prognostic models, our risk signature had the highest AUC values (Supplementary Figure S4). As shown in Table 4, only our risk signature had the prognosis capabilities in both univariate and multivariable Cox regressions.

**TABLE 4:** Comparisons between the risks core and other prognostic models using univariate and multivariate Cox regression analyses.

**Variable**	Univariate cox regression				Multivariate cox regression			
	HR	Lower 95% CI	Upper 95% CI	*p*-Value	HR	Lower 95% CI	Upper 95% CI	*p*-Value
Risk score	2.72	2.08	3.55	<0.001	2.61	1.92	3.50	<0.001
Fu et al	1.10	1.05	1.14	<0.001	1.04	0.98	1.00	0.19
Wu et al	1.97	1.40	2.77	<0.001	0.91	0.64	1.20	0.59

### External verification of the hypoxia-immune-based prognostic signature

**FIGURE 5:**
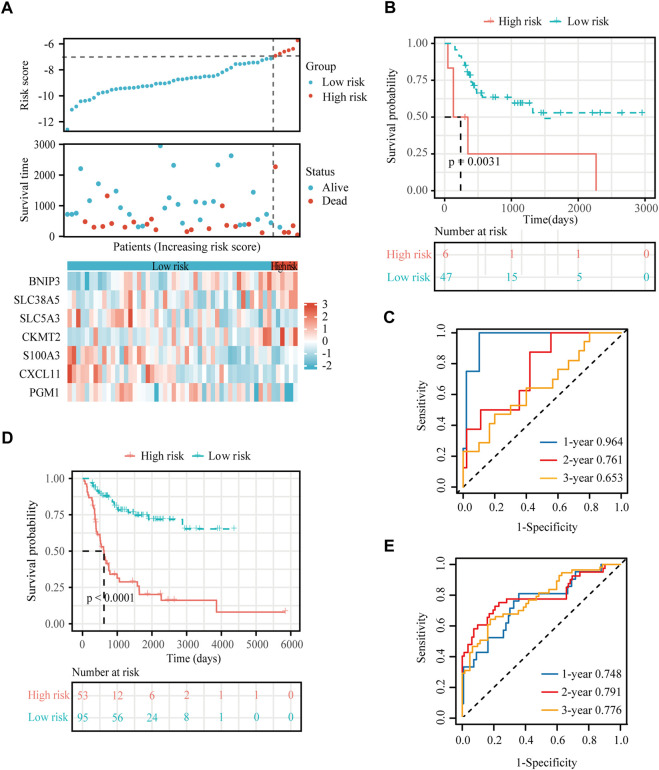
External verification for the hypoxia-immune-based prognostic signature. **(A)** Risk score, survival status and expression of the seven signature genes in the validation cohort from GSE21257 dataset. **(B)** K-M curve of overall survival for patients in the high-risk and low-risk groups in the validation cohort from GSE21257 dataset. **(C)** ROC curve for 1-, 2-, and 3-year overall survival in patients from the GSE21257 dataset. **(D)** K-M curve of overall survival for patients in the high-risk and low-risk groups in the combined dataset for aggregated analysis. **(E)** ROC curve for 1-, 2-, and 3-year overall survival in the combined dataset.

To verify the stability and reliability of the prognostic signature, we employed an independent cohort GSE21257 from GEO database for external verification. A total of 53 patients with complete survival information were enrolled in this validation cohort. The clinical information of all patients is shown in Table 5. Figure 5A showed the distribution of the risk score in GSE21257. K-M curves revealed that the overall survival of osteosarcoma patients in the high-risk group was significantly lower than the one in the low-risk group (log-rank test, *p* < 0.01) (Figure 5B). ROC curve analysis showed that AUC values for the 1-, 2- and 3-year survival rates were 0.964, 0.761 and 0.653, respectively (Figure 5C). Moreover, Cox regressions demonstrated that the risk score was significantly correlated with the overall survival of patients with osteosarcoma (Table 6).

**TABLE 5:** The clinical information of osteosarcoma patients in the verification cohort from GSE21257.

Characteristics	**Whole cohort (n = 53)**	**Low risk (n = 47)**	**High risk (n = 6)**
Age (year)			
≤16	25 (0.472)	21 (0.567)	4 (0.667)
>16	28 (0.528)	26 (0.433)	2 (0.333)
**Gender**			
Female	19 (0.358)	16 (0.340)	3 (0.500)
Male	34 (0.642)	31 (0.660)	3 (0.500)
**Metastasis**			
Yes	34 (0.642)	28 (0.596)	6 (1.000)
No	19 (0.358)	19 (0.404)	0
**Vital status**			
Alive	30 (0.566)	29 (0.617)	1 (0.167)
Dead	23 (0.434)	18 (0.383)	5 (0.833)
**Tumor locations**			
Leg/foot	44 (0.830)	41 (0.872)	3 (0.500)
Arm/hand	8 (0.151)	6 (0.128)	2 (0.333)
Unknown	1 (0.190)	0	1 (0.167)
**Risk group**			
High	6 (0.295)	0	6 (1.000)
Low	47 (0.705)	47 (1.000)	0

**TABLE 6:** Comparisons between the risk score and other four variables, including age, metastasis, gender, and primary tumor site in the GSE21257 dataset using univariate and multivariate Cox regression analyses.

**Variable**	**Univariate cox regression**					**Multivariate cox regression**			
	HR	Lower 95% CI	Upper 95% CI	*p* **-Value**		HR	Lower 95% CI	Upper 95% CI	*p*-Value
Risk score	1.44	1.00	2.08	<0.05		1.46	1.01	2.12	<0.05
Age	1.01	0.98	1.04	0.60		1.02	0.98	1.05	0.38
Gender	0.71	0.30	1.70	0.45		0.70	0.28	1.75	0.45
Tumor location	0.94	0.65	1.36	0.76		0.90	0.62	1.30	0.59

Next, a newly developed online tool, GEOexplorer, was applied to merge the training and validation cohorts for aggregate analysis. After combination, the new risk score of each patient was calculated. Patients were assigned to high-risk (n = 53) and low-risk (n = 95) groups based on the best optimal cutoff value of the risk score. K-M curves demonstrated that the overall survival of osteosarcoma patients in the high-risk group was remarkably lower than the one in the low-risk group (log-rank test, *p* < 0.0001) (Figure 5D). ROC curve analysis revealed that AUC values for the 1-, 2- and 3-year survival rates were 0.748, 0.791 and 0.776, respectively (Figure 5E).

Altogether, these results confirmed that our prognosis signature base on the seven genes BNIP3, SLC38A5, SLC5A3, CKMT2, S100A3, CXCL11, and PGM1 is valid and reproducible.

### Construction and calibration of an integrated nomogram

**FIGURE 6:**
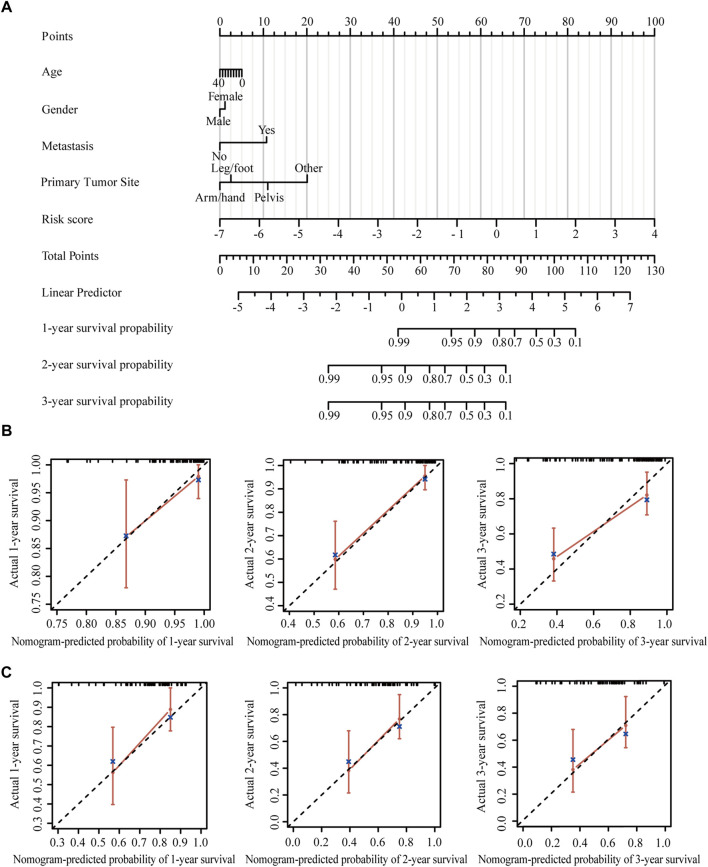
Construction and validation of the nomogram. **(A)** Nomogram to predict the 1-, 2-, and 3-year overall survival in patients from the TARGET dataset. **(B)** Calibration curves of the nomogram in patients from the TARGET dataset. **(C)** Calibration curves of the nomogram in patients from the GSE21257 dataset.

Nomogram provides a quantitative method for predicting the probability of patients’ overall survival, which could then be used in clinical practice. Based on the results of the multivariate Cox regression analysis, a nomogram was integrated with the hypoxia-immune-related risk signature together with other clinical features (Figure 6A). Every patient was assigned with a total points value by adding the points for each prognostic specific parameter. Higher total points corresponded to a worse clinical outcome of patients. The c-index value of the nomogram was 0.82, indicating a satisfactory overlap with actual observations. In addition, we also portrayed the corresponding calibration curves in 1, 2, and 3 years for internal and external verification. As shown in Figures 6B,C, the prediction lines of the nomogram for 1-, 2- and 3-year survival probability in the TARGET dataset and the GSE21257 dataset were quite close to the ideal performance (45° dotted line), indicating a high accuracy of the nomogram. Furthermore, ROC curves also supported the good predictive ability and accuracy of the nomogram for survival probability (Supplementary Figures S5A,B). Therefore, this nomogram, based on hypoxia-immune-related gene signature, might be used to predict the prognosis of osteosarcoma patients in clinical practice.

### Immune landscape between high- and low-risk groups of osteosarcoma patients

**FIGURE 7:**
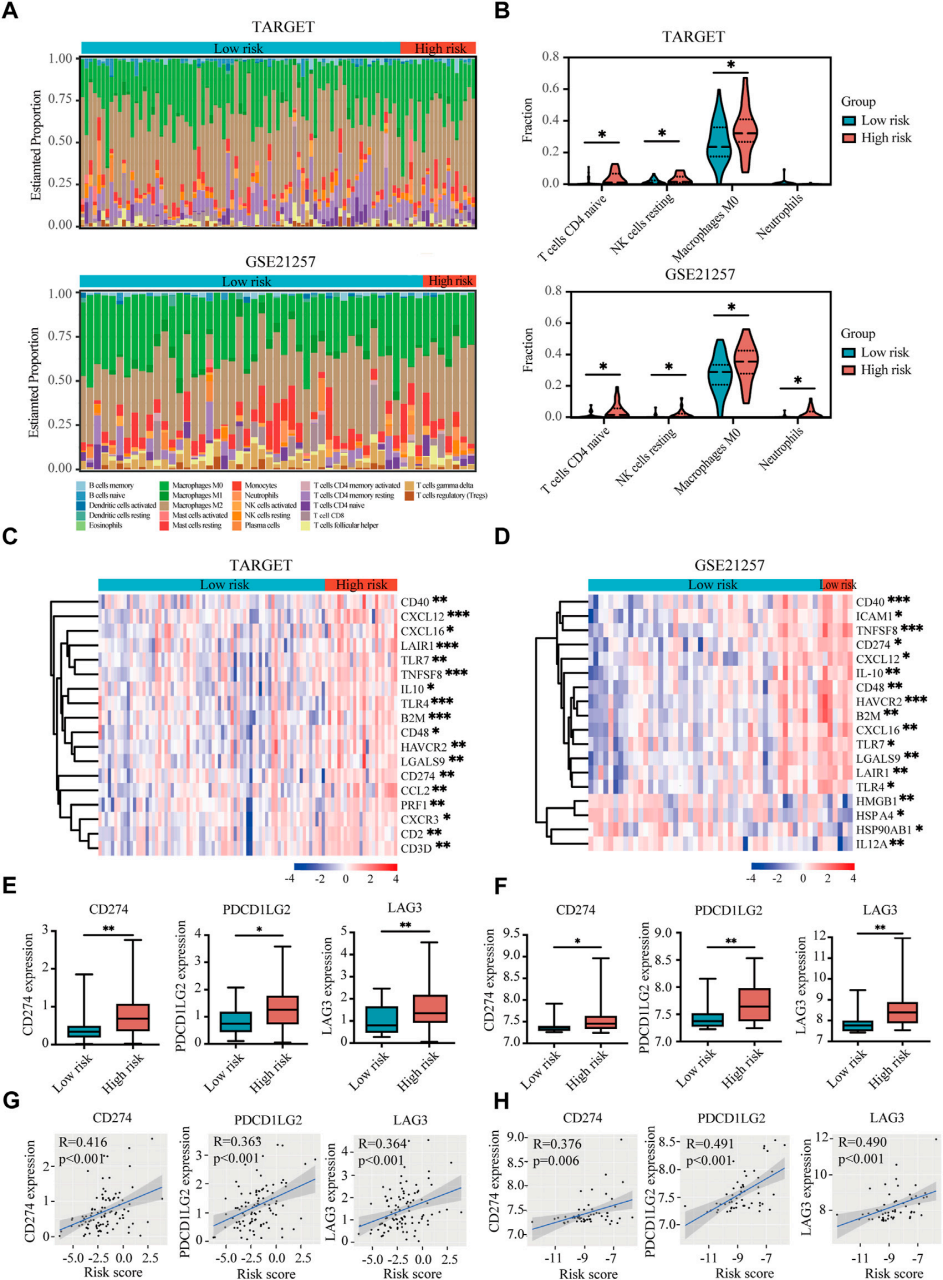
Immune landscape and immunosuppressive microenvironment between high-risk and low-risk groups of osteosarcoma patients. **(A)** Relative proportion of immune infiltration of 22 immune cells in the high-risk and low-risk groups in the TARGET and GSE21257 datasets. **(B)** Violin plots showing the significantly differences of immune cells between high-risk and low-risk groups in the TARGET and GSE21257 datasets. **(C,D)** Heatmaps of the immunosuppressive genes in the high-risk and low-risk groups in the TARGET **(C)** and GSE21257 datasets **(D)**. **(E,F)** Expressions of three immune checkpoint genes (CD274), PDCD1LG2, and LAG-3) in high-risk and low-risk groups in the TARGET **(E)** and GSE21257 datasets **(F)**. **(G,H)** Correlation between expressions of immune checkpoint genes and risk score in the TARGET **(G)** and GSE21257 datasets **(H)**. *p < 0.05, **p < 0.01, ***p < 0.001.

The tumor microenvironment regulates the antitumor immune responses by suppressing immune surveillance and promoting immune escape (Noman et al., 2015; Vaupel and Multhoff, 2017). However, the hypoxia-immune-related prognostic signature in the immune landscape was explored until now. CIBERSORT was used to identify the difference of 22 immune cells between high-risk and low-risk patients with osteosarcoma. In both TARGET and GSE21257 datasets, osteosarcoma patients with high risk had prominently higher proportions in immunosuppressive cells (such as naive CD4^+^ T cells, resting NK cells, M0 macrophages, and neutrophils) (Figures 7A,B). Thus, our prognostic signature might be highly associated with an immunosuppressive microenvironment.

### Immunosuppressive microenvironment between high- and low-risk groups of osteosarcoma patients

With the help of the Tracking Tumor Immunophenotype database (TIP; http://biocc.hrbmu.edu.cn/TIP/index.jsp) (Xu et al., 2018), we explored the expression of immunosuppressive genes in high- and low-risk groups of osteosarcoma patients. As shown in Figures 7C,D, most of these immunosuppressive genes were highly expressed in the high-risk group in both TARGET and GSE21257 datasets.

Next we analyzed three immune checkpoint genes, including PDL1 (CD274), PDL2 (PDCD1LG2), and LAG3, which were significantly upregulated in high-risk group and positively associated with risk score in both TARGET and GSE21257 datasets (Figures 7E–H).

### Experimental verification of the hypoxia-immune-based prognostic signature

**FIGURE 8:**
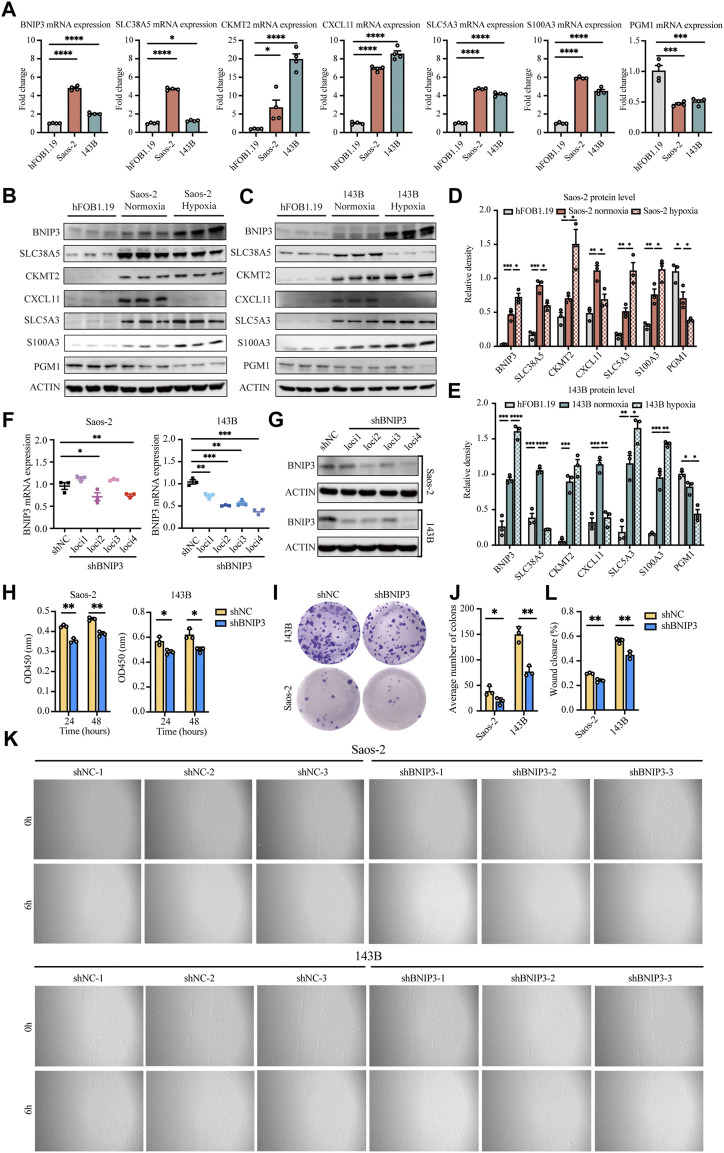
Validation of seven signature genes at mRNA and protein levels and functional analysis of BNIP3 *in vitro*. **(A)** RT-PCR for detecting the expression of seven genes in Saos-2 and 143B osteosarcoma cells and hFOB1.19 cells. **(B,D)** Western blot and quantification for the expression of the seven genes in Saos-2 cells (under hypoxic or normoxic conditions) and hFOB1.19 cells. **(C,E)** Western blot and quantification for the expression of the seven genes in 143B cells (under hypoxic or normoxic conditions) and hFOB1.19 cells. **(F)** RT-PCR for verifying the expression of BNIP3 in Saos-2 and 143B cells following transfection with its specific shRNAs. **(G)** Western blot for verifying the expression of BNIP3 in Saos-2 and 143B cells following transfection with its specific shRNAs. **(H)** CCK8 assay of detecting the proliferation ability of Saos-2 and 143B cells after BNIP3 knockdown. **(I,J)** Colony formation for measuring the proliferation capacity of Saos-2 and 143B cells after BNIP3 knockdown. **(K,L)** Wound healing assay for detecting the migration ability of Saos-2 and 143B cells after BNIP3 knockdown. *p < 0.05, **p < 0.01, ***p < 0.001.

The expression of BNIP3, SLC38A5, CKMT2, CXCL11, SLC5A3, S100A3, and PGM1 genes was verified in osteosarcoma cells (Saos-2 and 143B) and normal osteoblasts (hFOB1.19). Our results confirmed that BNIP3, SLC38A5, CKMT2, CXCL11, SLC5A3, and S100A3 displayed higher expression, while PGM1 showed lower expression in Saos-2 and 143B cells compared with hFOB1.19 cells (Figures 8A–E), indicating that the above signature genes might participate in the progression of osteosarcoma. Additionally, BNIP3, CKMT2, SLC5A3, and S100A3 were significantly upregulated, while SLC38A5, CXCL11, and PGM1was prominently downregulated in Saos-2 and 143B cells under hypoxic condition, compared with normoxic condition (Supplementary Figure S6). These results confirmed that those genes are regulated under an hypoxic environment.

### Silencing BNIP3 attenuates proliferation and migration of osteosarcoma cells

The biological functions of BNIP3 in tumor development were further investigated through knockdown experiments *in vitro*. BNIP3 knockdown efficacy was confirmed by RT-PCR and western blot in Saos-2 and 143B osteosarcoma cells (Figures 8F,G). Thereafter, experiments were performed with shBNIP3 loci4 in both Saos-2 and 143B cells. Cell proliferation assay showed that BNIP3 knockdown prominently attenuate the proliferation of osteosarcoma cells (Figures 8H–J). Cell migration analysis further demonstrated that BNIP3 knockdown significantly decreased the migration of osteosarcoma cells (Figures 8K,L).

## Discussion

Osteosarcoma is a highly aggressive bone tumor with an elevated tendency of metastasis. Although there are multiple advances in comprehensive therapies, the prognosis of osteosarcoma patients is still poor. Therefore, exploiting reliable prognostic signatures for osteosarcoma are sorely needed. With the development of bioinformatics and next-generation sequencing technology, numerous aberrantly expressed oncogenes have been detected and could be united as prognostic signatures in osteosarcoma (Xiao et al., 2020; Zhang et al., 2020; Fu et al., 2021; Jiang H et al., 2021). However, these prognostic signatures based on a single tumor characteristic were unable to reflect the disease characteristics of osteosarcoma adequately. To our knowledge, our research is the first to establish a prognostic signature based on two major tumor characteristics: hypoxia and immune response, which can reflect the hypoxia and immune status in osteosarcoma simultaneously.

In the present study, a total of 95 patients with osteosarcoma from the TARGET database were included in the training cohort to explore the potential value of a combined hypoxia and immune gene signature for the prognosis of osteosarcoma. By using the ssGSEA method and the ESTIMATE algorithm, we evaluated the hypoxia and immune infiltration status within the osteosarcoma microenvironment to obtain genes that are individually related to hypoxia and the immune state. Beside, another osteosarcoma dataset consisting of 36 freshly frozen paired samples (18 tumor and 18 non-tumor) further confirmed that hypoxia is a hallmark of osteosarcoma. We divided osteosarcoma patients into different groups according to an overlapping hypoxia and immune infiltration status and thereby successfully assigned several hypoxia-immune-related genes. Lasso regression model and stepwise Cox proportional hazards regression model were used to screen for the most robust genes to establish a prognostic signature. In addition, a formula for the calculation of the hypoxia-immune-based prognostic risk score. Then patients were divided into high- and low-risk groups according to the risk scores. Survival analysis demonstrated that high-risk patients had worse outcomes and the ROC curve analysis showed that the prognostic signature is robust and reliable. Subsequently, the capacity of this signature was validated in an independent cohort from the GEO database (GSE21257) and again after merging the TARGET and GSE21257 datasets. Importantly, we demonstrated that the risk score was an independent prognostic factor for osteosarcoma in both training and validation cohorts. The K-M plots of patients in different subgroups also showed that the high-risk patients lived for a shorter amount of time, suggesting the extensive applicability of the hypoxia-immune-based gene signature. Moreover, the nomogram including our prognostic signature (risk score) and certain other clinical parameters (age, gender, metastasis, and primary tumor site) was constructed to quantify the survival probability of osteosarcoma patients. Both calibration plots and ROC curves indicated the stable performance of the nomogram, which further supported the reliability of our prognostic signature. As a conclusion, the hypoxia-immune-based gene signature turned out to be a convincible biomarker for prognosis and might be used in the future for survival risk stratification and personalized management in osteosarcoma.

Immune cells are the main components of the tumor microenvironment, and their differentiation and function are often affected by the hypoxic microenvironment. In this study, we identified 688 DEGs related to hypoxia in the TARGET database. Enrichment analysis of these DEGs indicated that hypoxia is involved in several immune-associated biological processes, such as leukocyte migration and myeloid cell differentiation. Accumulating evidence strongly suggests that HIF-1α plays a major role in the regulation of immune cell function within the tumor microenvironment (Filippi et al., 2014; Liu et al., 2014). Filippi et al. reported an enhanced migratory capability of human monocyte-derived dendritic cells under hypoxic conditions (Filippi et al., 2014). Specifically, HIF-1α and PI3K/Akt signaling are responsible for hypoxia-induced activation of dendritic cells, sustaining their functions. Furthermore, HIF-1α induced by hypoxia has been implicated in the rapid differentiation of MDSCs into TAMs in the tumor microenvironment (Corzo et al., 2010). Sirtuin 1 (Sirt1) was found to be the main driver in the regulation of MDSCs differentiation through HIF-1-mediated glycolytic metabolic reprogramming and has an impact on MDSC functions in both immune suppression and promotion of tumor progression (Liu et al., 2014). These results highlight the tight link between hypoxia and immune cell infiltration in the osteosarcoma microenvironment.

Our results found significantly higher proportions of naive CD4^+^ T cells, resting NK cells, M0 macrophages, and neutrophils in high-risk patients with osteosarcoma, suggesting a signature of an immunosuppressive microenvironment. NK cells played an essential role in anticancer immunity, however, the activity of NK cells in the tumor microenvironment is often repressed (Stojanovic et al., 2013). TAMs and neutrophils have been reported to enhance tumor cell invasion, metastasis and angiogenesis, while suppressing tumor immune surveillance (Kim and Bae, 2016). Moreover, immune checkpoints are crucial in carcinogenesis for enhancing the antitumor effects of immune cells. PDL1/2 and LAG3 are highly effective against advanced malignancies with fewer side effects than conventional therapies (Ren et al., 2019; Jiang F et al., 2021; Luke et al., 2022). Here, the above three immune checkpoints were meaningfully upregulated in the high-risk group and positively related to risk scores, confirming an immunosuppressive microenvironment in high-risk patients with osteosarcoma.

The prognostic signature we constructed contains the following seven genes: BNIP3, SLC38A5, SLC5A3, CKMT2, S100A3, CXCL11 and PGM1. Our experimental work revealed that BNIP3, SLC38A5, CKMT2, CXCL11, SLC5A3, and S100A3 were expressed at higher levels, whereas PGM1 was expressed at lower levels in osteosarcoma cells compared with normal osteoblasts, highlighting the significance of the above genes in the occurrence and development of osteosarcoma. Important roles of these signature genes have been reported previously in multiple types of cancers. BNIP3, which functions downstream of HIF-1α, plays a crucial role in carcinogenesis (Vijayalingam et al., 2010). Similar to our findings in osteosarcoma, increased BNIP3 level has been reported to be correlated with an aggressive tumor phenotype and a poor prognosis in a number of cancers, including breast cancer, non-small cell lung cancer, and uveal melanoma (Giatromanolaki et al., 2004; Chourasia et al., 2015; Jiang et al., 2018). Our *in vitro* models confirmed that BNIP3 knockdown significantly inhibited the proliferation and migration ability of osteosarcoma cells. Vianello et al. indicated that BNIP3 could be a potential target to overcome the treatment resistance in osteosarcoma. By inhibiting BNIP3-mediated mitophagy, the resistance to cisplatin observed in osteosarcoma could be reduced (Vianello et al., 2022).

SLC38A5, an amino acid transporter, is upregulated in a variety of cancers to mediate the influx of glutamine, serine, glycine, and methionine into cancer cells (Girardi et al., 2020). It plays a critical role in promoting the survival and proliferation of cancer cells and represents a novel target for cancer therapy (Sniegowski et al., 2021). In theory, small molecules with high affinity and selectivity to inhibit its transport function could potentially have efficacy as anticancer drugs. Published reports have demonstrated the therapeutic utility of α-methyl-l-tryptophan as a fairly selective inhibitor of SLC6A14 in the treatment of breast cancer (Karunakaran et al., 2011), pancreatic cancer (Coothankandaswamy et al., 2016), and colon cancer (Sikder et al., 2020). However, SLC38A5 as a drug target for osteosarcoma would require further investigations.

CKMT2, also known as s-MtCK, belongs to the creatine kinase isoenzyme family. The overexpression of CKMT2 has been reported in malignant tumors (Pratt et al., 1987). This isoenzyme may represent a tumor marker (Fusae et al., 1984). Wang H et al. have shown that CKMT2 might serve as a key regulating factor participating in osteosarcomagenesis (Wang et al., 2017).

Regarding CXCL11, its expression was positively correlated with prolonged overall survival in lung cancer (Cao et al., 2021) and colon adenocarcinoma patients (Gao et al., 2019). CXCL11 is a major chemoattractant for effector T cells, thus CXCL11-dependent therapy may be a potential approach for cancer treatment (Colvin et al., 2004). For instance, Liu et al. was able to attract T cells and NK cells into the tumor microenvironment and enhance their therapeutic efficacy by overexpressing CXCL11 with an oncolytic vaccinia viru (Liu et al., 2016).

Since the role of SLC5A3 and S100A3 has been scarcely studied in osteosorcoma, the last gene from our signature pattern that is worth mentioning is PGM1. It belongs to the phosphohexose mutase family and acts as an important regulator in the glucose metabolism (Jin et al., 2018). PGM1 depletion reduces the glycogen content and the rates of glycogenolysis and glycogenesis, subsequently suppressing tumor cells proliferation (Bae et al., 2014). Collectively, the novel gene signature identified in this study could provide hitherto unexplored therapeutic targets and directions for the elucidation of molecular mechanisms in osteosarcoma, however, further investigations would be required to experimentally confirmed our *in silico* findings.

Some limitations should be noted in this study. Firstly, the number of osteosarcoma samples is relatively small, because of the low incidence and lack of studies in this field. Further studies with a higher sample number would be needed to better evaluate the performance of our signature. Secondly, the datasets used in this study were based on retrospective investigation. More prospective studies are needed to further confirm the prognostic value of hypoxia-immune-based gene signature in osteosarcoma.

## Conclusion

In summary, we constructed a new hypoxia/immune cell infiltration axis that correlated with the prognosis of osteosarcoma patients. Thereafter, a novel prognostic signature based on hypoxia- and immune-related genes was developed and validated, which has favorable prognostic prediction performance and promising clinical application in osteosarcoma.

## Data Availability

The datasets presented in this study can be found in online repositories. The names of the repository/repositories and accession number(s) can be found in the article/Supplementary Material.
