# Supplementary material for: Hypoxia-immune-related microenvironment prognostic signature for osteosarcoma
Source: Front Cell Dev Biol. 2022 Dec 12;10:974851. doi: 10.3389/fcell.2022.974851 (PMC9791087; doi:10.3389/fcell.2022.974851)
Supplement: Supplementary file 1 [file DataSheet1.PDF]

Supplementary Material

Supplementary Figures

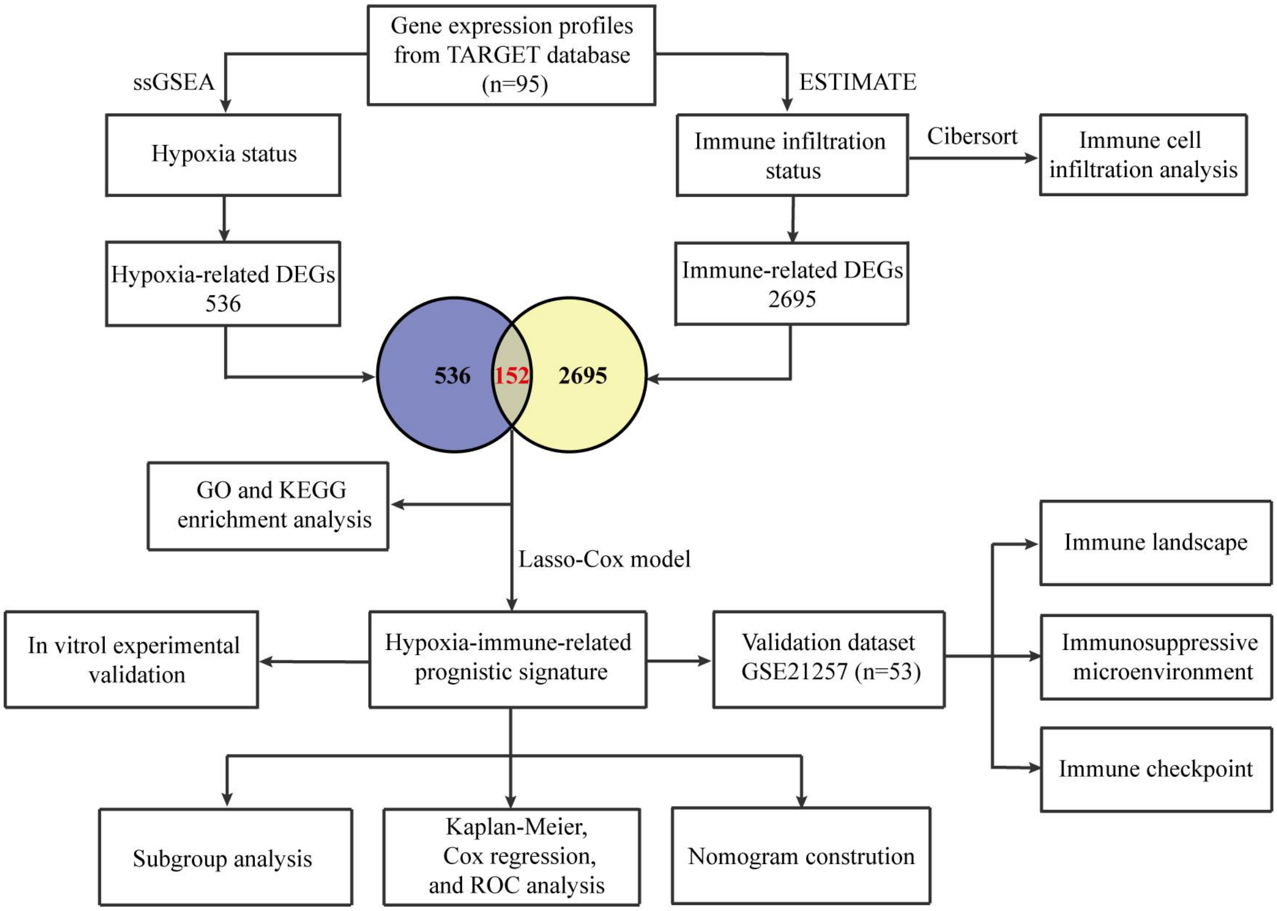

Supplementary Figure 1. The flowchart of the study.

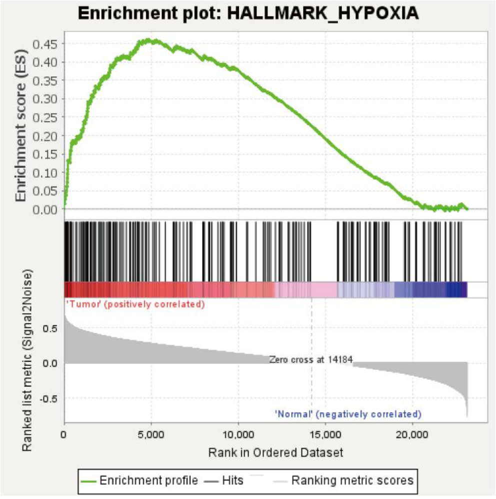

Supplementary Figure 2. GSEA analysis of hypoxia pathway between tumoral bone samples and non-tumoral paired samples in the GSE99671 dataset.

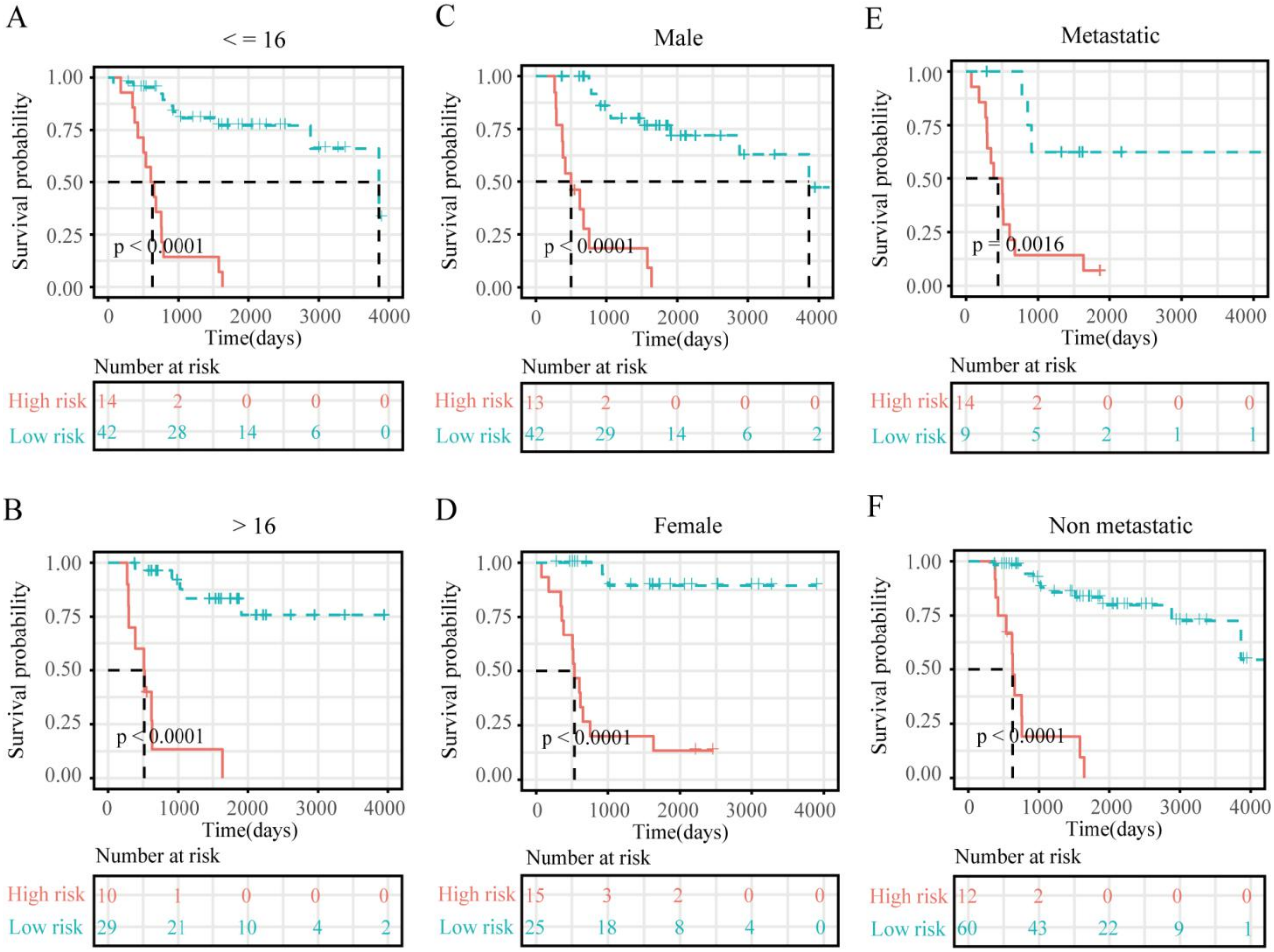

Supplementary Figure 3. Prognostic significance of hypoxia-immune-based gene signature in osteosarcoma patients with different clinical features. (A) Age < = 16, (B) Age > 16, (C) Male, (D) Female, (E) Metastasis, and (F) Non metastasis.

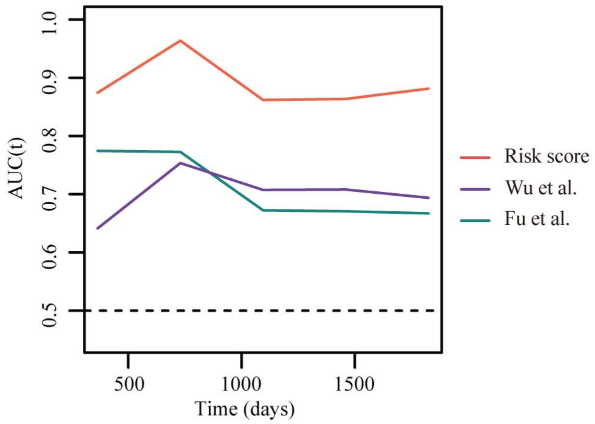

Supplementary Figure 4. Comparisons of AUC values among different gene signatures of osteosarcoma.

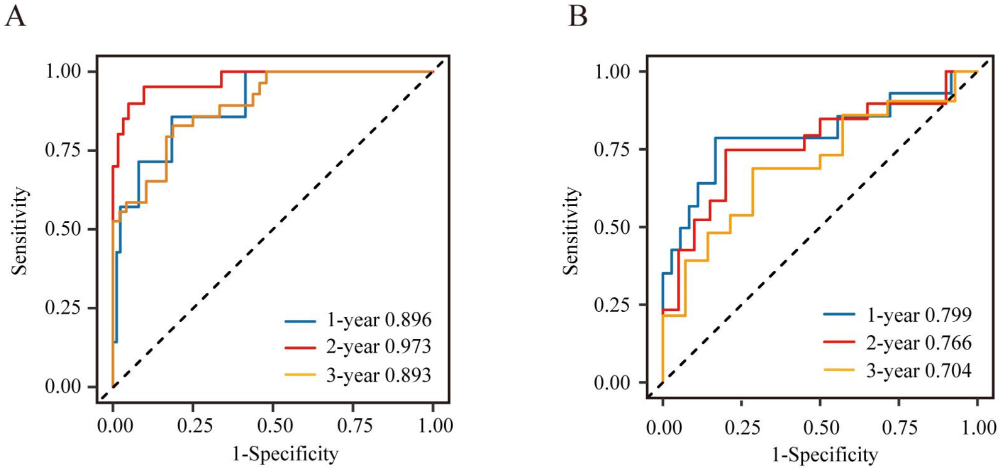

Supplementary Figure 5. ROC curves of nomogram for 1-, 2-, and 3-year overall survival in patients from the TARGET dataset (A) and the GSE21257 dataset (B).

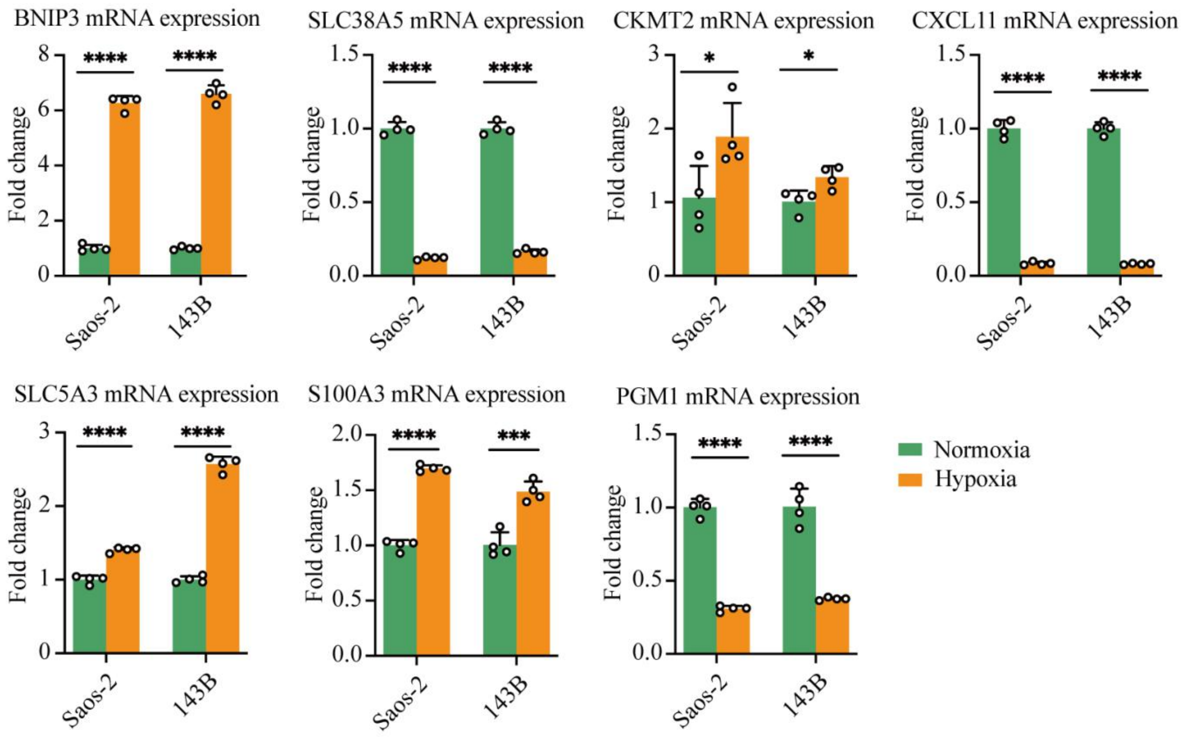

Supplementary Figure 6. RT-PCR for measuring the expression of 7 genes in osteosarcoma cells (Saos-2 and 143B) under hypoxic or normoxic conditions.
